# Expression of Cathepsin D in early-stage breast cancer and its prognostic and predictive value

**DOI:** 10.1007/s10549-024-07293-y

**Published:** 2024-04-05

**Authors:** Inas Alhudiri, Christopher Nolan, Ian Ellis, Adam Elzagheid, Andrew Green, Caroline Chapman

**Affiliations:** 1 Breast Pathology Research Group, Nottingham Breast Cancer Research Centre, Biodiscovery Institute, Faculty of Medicine, The University of Nottingham, Nottingham, UK; 2 Genetic Engineering Department, Libyan Biotechnology Research Centre, Tripoli, Libya; 3 Eastern Bowel Cancer Screening Hub, Nottingham University Hospitals, NHS Trust, Nottingham, UK

**Keywords:** Breast cancer, Cathepsin D, Immunohistochemistry, Tissue microarray, Prognosis

## Abstract

**Purpose:**

Cathepsin D is a proteolytic enzyme that is normally localized in the lysosomes and is involved in the malignant progression of breast cancer. There are conflicting results regarding Cathepsin D significance as prognostic and predictor marker in breast cancer. This study aimed to evaluate the expression and prognostic significance of Cathepsin D in early-stage breast cancer.

**Methods:**

Expression of Cathepsin D was assessed by immunohistochemical staining of tissue microarrays, in a large well-characterized series of early-stage operable breast cancer (*n* = 954) from Nottingham Primary Breast Carcinoma Series between the period of 1988 and 1998 who underwent primary surgery. Correlation of Cathepsin D expression with clinicopathological parameters and prognosis was evaluated.

**Results:**

Cathepsin D expression was positive in 71.2% (679/954) of breast cancer tumours. Positive expression of Cathepsin D was significantly associated with high histological grade (*p* = 0.007), pleomorphism (*p* = 0.002), poor Nottingham Prognostic Index (NPI) score (*p* < 0.002), recurrence (*p* = 0.005**)** and distant metastasis (*p* < 0.0001). Kaplan–Meier analysis showed that Cathepsin D expression was significantly associated with shorter breast cancer-specific survival (*p* = 0.001), higher risk of recurrence (*p* = 0.001) and distant metastasis (*p* < 0.0001). ER-positive tumours expressing Cathepsin D and treated with tamoxifen demonstrated a significantly higher risk of distant metastasis.

**Conclusion:**

Cathepsin D expression significantly predicts poor prognosis in breast cancer and is associated with variables of poor prognosis and shorter outcome. The strong association of Cathepsin D with aggressive tumour characteristics and poor outcomes warrants further research of its potential as a therapeutic target The results also suggest a possible interaction between Cathepsin D and tamoxifen therapy in ER-positive breast cancer which needs further investigation to elucidate the underlying mechanisms.

## Introduction

Cathepsin D is an aspartic endoproteinase that is localized in the lysosomes and extracellular matrix and is involved in the malignant progression of breast cancer [1]. It catalyses proteins into several polypeptide fragments that digest other lysosomal endopeptidases and exopeptidases [2]. Cathepsin D is synthesized on the rough endoplasmic reticulum as preproprotein (412 amino acids, 52 kDa) that is subjected to a series of proteolytic cleavages during biosynthesis to produce the mature enzyme [3].

Importance of Cathepsin D in breast cancer was first emerged by the studies of proteins whose expression is regulated by oestrogen in breast cancer [4]. Cathepsin D has long been recognized as an oestrogen-regulated protein [5]. Oestrogen regulates Cathepsin D through binding to oestrogen response elements (EREs) and modulating the transcription of Cathepsin D [6].

Cathepsin D induces cell proliferation, metastasis, tumour invasion, angiogenesis and apoptosis in cancer and stromal cells [7–10]. As a consequence, several studies have been undertaken to evaluate its clinical significance in breast cancer [11]. However, Cathepsin D has not been recommended for clinical use by American Society of Clinical Oncology (ASCO) because of insufficient evidence [12].

This study aimed to address the gap in the literature on the prognostic value of Cathepsin D expression in breast cancer patients. We analysed a large and well-characterized cohort of breast cancer cases with complete data on classical prognostic factors and relevant molecular markers, such as oestrogen receptor (ER), progesterone receptor (PgR) and HER2 as well as Cathepsin D’s role in response to tamoxifen therapy. The results described in this paper detailed findings in the whole series as well as selected patient subgroups according to their ER, HER2 and Cathepsin D status.

## Materials and methods

### Patients and tissue specimens

Tissue microarrays (TMAs) were constructed from 954 cases of primary operable invasive breast carcinoma obtained from Nottingham Tenovus Primary Breast Carcinoma Series. This series is a well-characterized case of primary breast carcinoma of long-term follow-up with clinical and pathological data including tumour type, tumour size, histological grade, vascular invasion and Nottingham Prognostic Index (NPI). Information on locoregional recurrence, distance metastasis, nodal status, survival and therapy was prospectively collected [13, 14]. Data on a wide range of breast cancer biomarkers were also available including ER-PgR and HER2 [15].

Breast cancer-specific survival (BCSS) was defined as the time interval (in months) from the date of the primary surgery to the time of death from breast cancer, and disease-free interval (DFI) was defined as the length of time (in months) from the date of the primary surgical treatment to the first locoregional recurrence or distant metastasis. Patients were divided according to their NPI score into three prognostic groups: good (≤ 3.4), moderate (3.41–5.4) and poor (> 5.4) [16]. The median follow-up time was 147 months (range 1–243). Clinicopathological and patient characteristics are listed in Table 1.

**Table 1:** Patient characteristics

Clinicopathological criteria	No. of patients
Age	
≤ 50 years	320
≥ 50 years	634
Local recurrence	411
Distant metastasis	319
Lymph node stage	
1 (negative)	553
2 (1–3)	289
3 (> 3)	87
Local radiotherapy	
No	373
Yes	525
Radiotherapy to LN	
No	698
Yes	200
Tumour size	
< 1.5 cm	201
> 1.5 cm	728
Definite vascular invasion	313
Endocrine therapy	300
Chemotherapy	178
Grade	
1	144
2	282
3	503
NPI score	
Good	235
Moderate	517
Poor	175

The patients were managed in a uniform way according to their hormonal status and NPI score; those within the good prognostic group received no adjuvant therapy, those with an NPI score > 3.4 received Tamoxifen if ER positive (± Zoladex if pre-menopausal) or classical cyclophosphamide, methotrexate and 5-fluorouracil if ER negative and suitable for chemotherapy [17].

Ethical approval for this study was granted by Nottingham Research Ethics committee 2 under the title of “Development of a molecular genetic classification of breast cancer”.

### Immunohistochemical staining

Immunohistochemical staining was performed using Dako REAL™ EnVision™ Detection System (Invitrogen). A monoclonal antibody for Cathepsin D (Millipore Mouse monoclonal antibody #MAB422) was used for immunohistochemically evaluation. After deparaffinization in xylene and rehydration through graded alcohol, sections were immersed in 1:10 citrate buffer pH 6.0 and microwaved for 20 min in order to retrieve antigenicity. Once the retrieval is complete, slides were rinsed with tris buffered saline (TBS) pH 7.6 and endogenous peroxidase activity was inhibited by applying 100 µl peroxidase block (Dako Real peroxidise blocking solution, S2023) to the TMA sections for 5 min followed by TBS rinse. Sections were then treated with 100 µl Ultra V block (ThermoScientific TA-125-UB) to block non-specific staining by the primary antibody for 5 min at room temperature. The antibody was diluted to 1:100 optimal working dilution and incubated for 30 min at room temperature. After washing with TBS, all sections were then incubated with 100 μl of a secondary antibody (dextran coupled peroxidase molecules and goat anti-mouse/rabbit immunoglobulin; Dako REAL™ EnVision™/HRP, Rabbit/Mouse bottle A, K5007) for 30 min. Sections were washed in TBS and incubated with 100 μl of a freshly prepared solution of 3′3-diaminobenzidine (Dako Envision Kit, Bottle B and C, K5007) for 5 min and repeated once. After rinsing in TBS three times, sections were then counterstained with haematoxylin (Dako Real Automation Haematoxylin, S3301) for 6 min. After washing in tap water, the sections were dehydrated in ethanol, cleared in xylene and mounted with DPX (BDH, Poole, UK). Formalin-fixed paraffin-embedded normal liver tissue was used as a positive control. We employed normal tissue within the same tumour sample as the negative control.

### Analysis of immunohistochemical staining

Immunoreactivity of Cathepsin D in the TMA cores was evaluated in a semi-quantitative way by assessing both percentages of cells stained and intensity of staining. Cytoplasmic staining of the TMA cores was measured using the modified Histo-score (H-Score) with a range from 0 to 300 scores. Only staining of the invasive malignant cells within the tissue cores was considered. TMAs were scored using high-resolution digital images (NanoZoomer; Hamamatsu Photonics, Welwyn Garden City, UK), at ×20 magnification, using a web-based interface (Distiller; Slidepath Ltd, Dublin, Ireland). All samples were scored by one observer (IA) and a proportion of these were scored twice on two separate assessments. Cores were only scored if tumour cells represented greater than or equal to 15% of the total core.

### Statistical analysis

Statistical analysis was performed using SPSS v24 statistical software. Chi-square (x^2^) analyses were used to test correlations between antigen expression and clinicopathological parameters, steroid receptors and biomarkers. Possible correlation between antigen expression levels and breast cancer-specific survival (BCSS) and disease-free interval was examined using Kaplan–Meier curves and differences between the curves were analysed using the log-rank test. Cox regression models were used for multivariate analysis to test the effect of antigen expression and clinicopathological parameters on disease-free survival and breast cancer-specific survival (BCSS) as well as its statistical independence. A *p*-value of < 0.05 was considered statistically significant. Data were dichotomized into two groups according to frequency distributions and Kaplan–Meier curves of the effect on BCSS and cut-off was chosen using X-tile Bioinformatics software [18].

## Results

### Evaluation of immunohistochemical analysis

**Fig.1:**
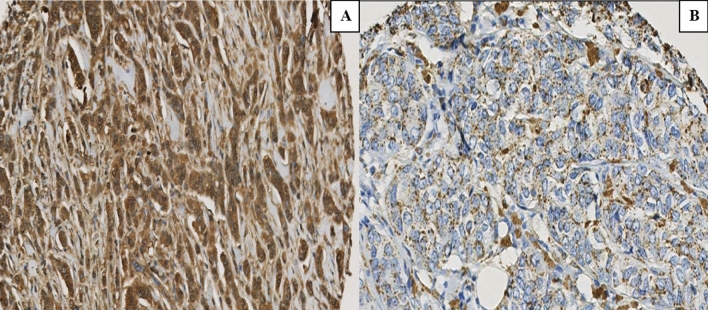
**A** Cathepsin D strong positive cytoplasmic staining, **B** Cathepsin D-negative/weak cytoplasmic staining

Immunohistochemical examination of breast carcinoma microarray sections revealed positive staining of Cathepsin D localized to the cytoplasm of invasive malignant cells. Most tumour cores were homogenously stained with Cathepsin D. A total of 80.4% of tumour cores showed different degrees of cytoplasmic staining to Cathepsin D while 19.6% did not show any cytoplasmic staining (Fig. 1). Negative Cathepsin D expression was defined as cytoplasmic H-score ≤ 30 and positive expression as cytoplasmic stained score > 30. Negative expression rate of Cathepsin D using the cut-off value was 275/954 (28.8%) while positive expression rate of Cathepsin D was 679/954 (71.2%).

### Correlation of Cathepsin D expression with clinicopathological parameters

Cathepsin D expression was significantly correlated with histological grade, pleomorphism, NPI, distant metastasis and recurrence (Table 2). Positive Cathepsin D expression was associated with high-grade tumours (*p* = 0.007), high pleomorphism score (*p* = 0.002), poor NPI score (*p* = 0.002), development of distant metastasis (*p* < 0.0001) and recurrence (*p* = 0.005). Cathepsin D expression was not associated with age at diagnosis, tumour stage, tumour size, nor vascular invasion. Positive expression of Cathepsin D was associated with invasive ductal, tubular mixed, atypical medullary and lobular mixed histological tumour types (*p* = 0.009) as shown in Table 3.

**Table 2:** Correlation of Cathepsin D expression with clinicopathological parameters

Parameter	Negative n = 275 (%)	Positive n = 679 (%)	*p*-Value
Grade			
1	53 (19.6)	91 (13.8)	0.007
2	92 (33.9)	190 (28.9)	
3	126 (46.5)	377 (57.3)	
Total	271	658	
LN stage			
1(negative)	176(64.9)	377(57.3)	0.084
2 (1–3)	75(27.7)	214(32.5)	
3(> 3)	20(7.4)	67(10.2)	
Total	271	658	
Tumour size			
< 1.5 cm	62(22.9)	139(21)	0.555
≥ 1.5 cm	209(77)	519(78.9)	
Total	271	658	
Tubules			
1	20 (7.6)	26(4)	0.091
2	85(32.3)	213(33.4)	
3	158(60)	399(62.5)	
Total	263	638	
Pleomorphism			
1	7(2.7)	6(0.9)	0.002
2	109(41.6)	203(31.9)	
3	146(55.7)	428(67.2)	
Total	262	637	
Distant metastases			
No	205(75.4)	424(62.7)	< 0.0001
Definite	67(24.6)	252(37.3)	
Total	272	676	
Nottingham Prognostic Index			
Good	84(31)	151(23)	0.002
Moderate	151(55.9)	366(55.7)	
Poor	35(13)	140(21.3)	
Total	270	657	
Vascular Invasion			
Negative	189(70.3)	427(64.7)	0.104
Probable	80(29.7)	233(35.3)	
Total	269	660	
Recurrence			
No	171(63)	352(53)	0.005
Yes	100(36.9)	311(46.9)	
Total	271	663	
Local radiotherapy			
No	123 (47.3)	250 (39.2)	0.025
Yes	137 (52.7)	388 (60.8)	
Radiotherapy to LN			
No	214 (81.7)	484 (76.1)	0.068
Yes	48 (18.3)	152 (23.9)	
Total	262	636

**Table 3:** Association of Cathepsin D expression in breast cancer with histological type

Tumour type	Negative (%)	Positive (%)
Invasive ductal/no special type	140 (24.5)	431(75.5)
Tubular mixed	50 (32.9)	102 (67.1)
Atypical medullary	6 (26.1)	17(73.9)
Classical lobular	20(46.5)	23(53.5)
Lobular mixed	8 (28.6)	20(71.4)
Mixed NST and lobular	16(47.1)	18(52.9)
Tubular	10(38.5)	16(61.5)
Mixed NST and A special type	8 (57.1)	6(42.9)
Mucinous	2 (33.3)	4(66.7)
Typical medullary	0	2(100)
Solid lobular	1 (50.0)	1(50.0)
Tubulo-lobular	2 (50)	2(50)
Invasive papillary	0	4(100)
Miscellaneous types	2 (50)	2(50)
*P* = 0.009		

*NST* stands for No special type

### Association of Cathepsin D with hormone receptor and HER2 status

Cathepsin D was highly associated with ER and HER2 status but not PgR. Tumours positive for Cathepsin D were associated with ER-positive (*p* = 0.006) and HER2-negative (*p* = 0.006) status. Tumours positive for both ER and PgR were also associated with positive Cathepsin D expression (*p* = 0.037). Cathepsin D immunoreactivity showed no correlation with triple negative status (*p* = 0.228). Correlation between Cathepsin D and hormone receptor status is summarized in Table 4.

**Table 4:** The relationship between Cathepsin D expression and other biomarkers

Patients		Negative for Cathepsin D (%)	Positive for Cathepsin D (%)	*p*-value
ER	Negative	59 (22.2)	202 (31.3)	0.006
	Positive	207 (77.8)	443 (68.7)	
	Total	266	645	
PgR	Negative	98 (38.4)	284 (44.3)	0.109
	Positive	157 (61.6)	357 (55.7)	
	Total	521	378	
HER2	Negative	242 (91.3)	547 (84.4)	0.006
	Positive	23 (8.7)	101 (15.6)	
	Total	265	648	
Triple negative	No	220 (83.0)	513 (79.5)	0.228
	Yes	45 (17.0)	132 (20.5)	
	Total	265	645	
ER-PgR status	Both absent	53 (21.0)	187 (29.8)	0.037
	ER absent	2 (0.8)	11 (1.8)	
	Both positive	155 (61.8)	342 (54.5)	
	ER only	41 (16.3)	87 (13.9)	
	Total	251	627	

### Association of Cathepsin D expression with patient outcome

**Fig.2:**
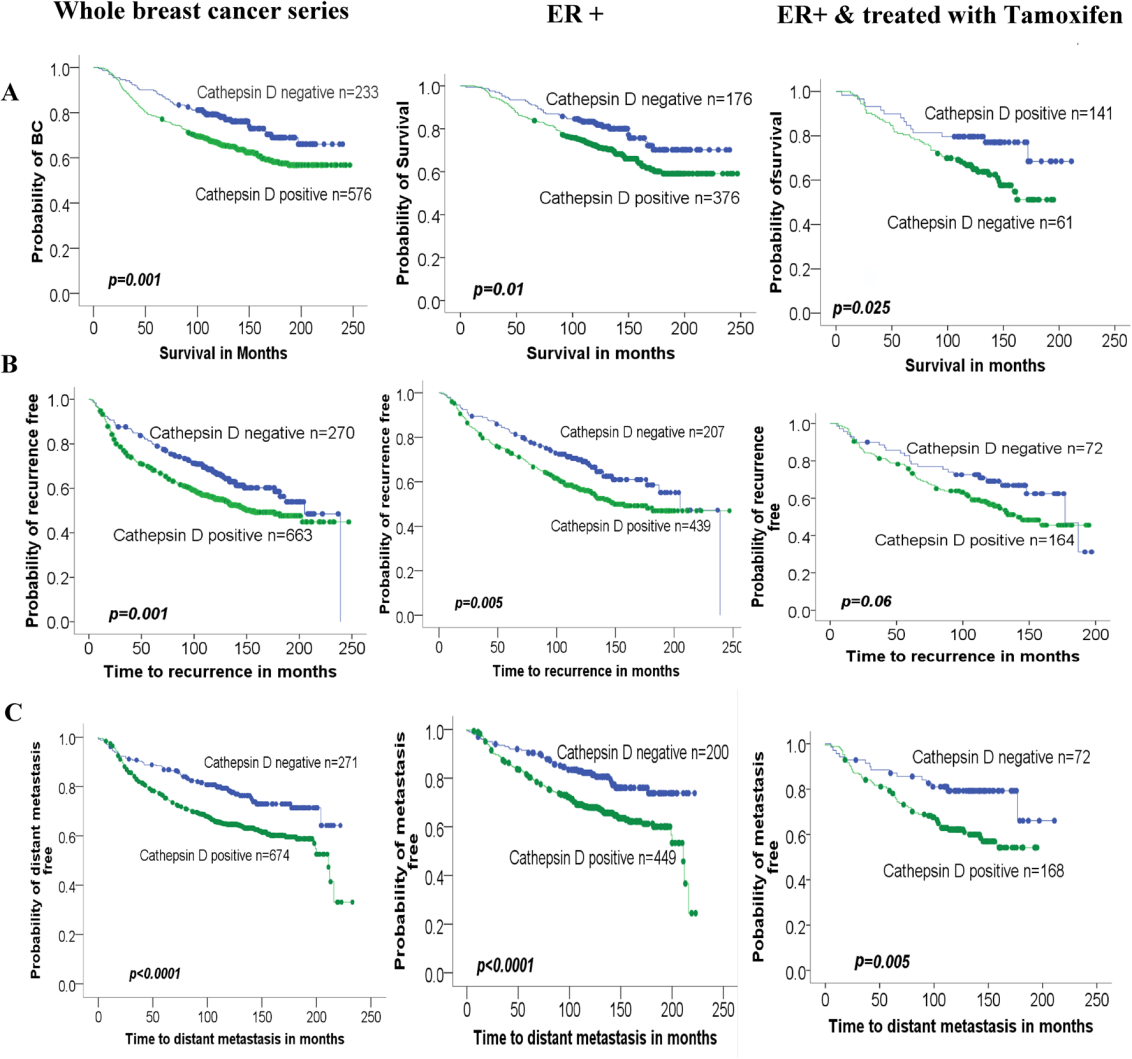
Kaplan–Meier analysis for Cathepsin D expression in correlation with patient outcome in the whole series, ER+ cancers and ER+ cancers on tamoxifen therapy for survival (**A**), local recurrence (**B**) and distant metastasis (**C**)

Kaplan–Meier survival analysis of breast cancer-specific survival (BCSS) showed significantly poor prognosis for Cathepsin D-expressing tumours compared with those negative for the biomarker (*p* = 0.001) (Fig. 2). In multivariate Cox-proportional hazards analysis, Cathepsin D expression was a significant prognosticator of BCSS (*p* = 0.021) independent of tumour grade, tumour size, lymph node stage and ER status (Table 5). However, expression of Cathepsin D was not significantly independent when HER2 was added to the regression model (*p* = 0.057).

**Table 5:** Multivariate Cox regression analysis of factors associated with BCSS

BCSS				BCSS (adding HER2)		
Variable	Hazard ratio	*p-*value	95% CI	Hazard ratio	*p-*value	95% CI
Tumour size	1.767	0.004	1.195–2.613	1.789	0.004	1.203–2.662
Tumour stage	1.779	< 0.0001	1.507–2.101	1.769	< 0.0001	1.496–2.091
Tumour grade	1.823	< 0.0001	1.460–2.277	1.722	< 0.0001	1.379–2.149
Cathepsin D	1.412	0.021	1.053–1.829	1.331	0.057	0.992–1.787
ER	0.819	0.174	0.626–1.072	0.874	0.335	0.666–1.148
HER2				1.724	< 0.0001	1.274–2.332

Univariate analysis showed that positive expression of Cathepsin D was strongly associated with development of distant metastasis (*p* < 0.0001) and recurrence (*p* = 0.001); (Fig. 2). In multivariate Cox-proportional hazards analysis, Cathepsin D expression was a significant prognosticator of distant metastasis and recurrence (*p* = 0.004 and *p* = 0.021 ,respectively) independently of tumour grade, size, lymph node stage, HER2 and ER status (Table 6).

**Table 6:** Multivariate Cox regression analysis of factors associated with DFI

Disease-free interval (metastasis)				Disease-free interval (recurrence)		
Variable	Hazard ratio	*p-*value	95% CI	Hazard ratio	*p-*value	95% CI
Tumour size	1.654	0.005	1.160–2.357	1.341	0.039	1.014–1.773
Tumour stage	1.990	< 0.0001	1.691–2.340	1.689	< 0.0001	1.458–1.957
Tumour grade	1.408	0.001	1.152–1.722	1.234	0.012	1.047–1.453
Cathepsin D	1.533	0.004	1.151–2.042	1.323	0.021	1.044–1.676
ER	0.923	0.556	0.707–1.205	1.001	0.996	0.785–1.275
HER2	1.496	0.008	1.111–2.015	1.361	0.029	1.032–1.795

### Prognostic significance of Cathepsin D expression as a function of the oestrogen receptor status and tamoxifen therapy

Patients were subdivided into ER-negative and ER-positive subsets and the clinical outcome in terms of BCSS and DFI was analysed according to Cathepsin expression using univariate Kaplan–Meier survival analysis (Fig. 2). In ER-positive tumours, patients with positive Cathepsin D expression showed significantly shorter BCSS than those negative for the marker (*p* = 0.01). In ER-positive tumours, patients with positive Cathepsin D expression showed significantly shorter disease-free interval (*p *= 0.005 for recurrence and *p* < 0.0001 for metastasis) than those negative for the marker. In ER-negative tumours, Cathepsin D expression was not associated with BCSS and DFI.

**Fig.3:**
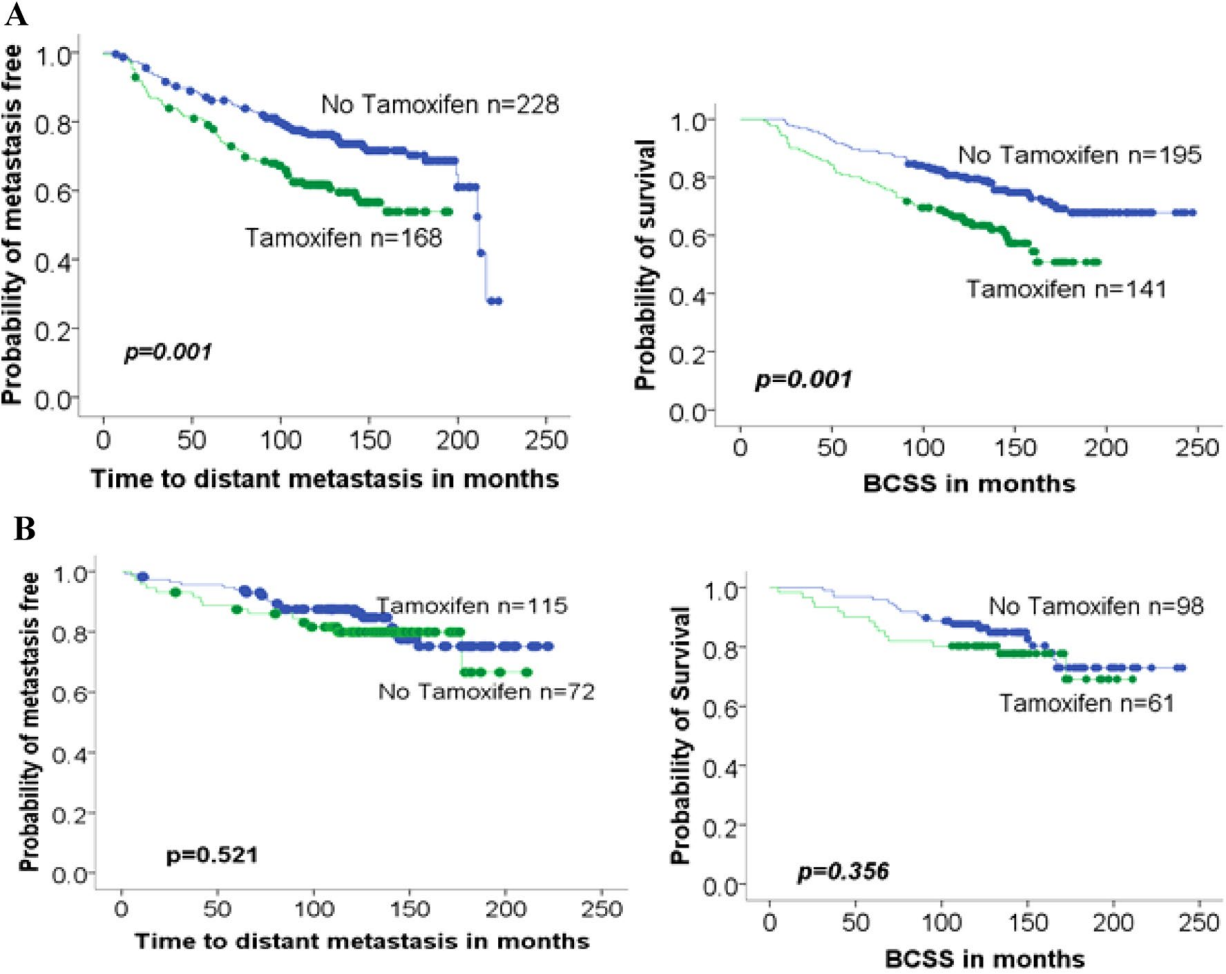
Association of Cathepsin D expression with tamoxifen treatment. **A** Cathepsin D-positive, ER-positive tumours. **B** Cathepsin D-negative, ER-positive tumours. Cathepsin D predicts the development of BCSS and distant metastasis in patients on tamoxifen

Patients with cancers expressing Cathepsin D exhibited significantly lower BCSS if they had received tamoxifen (*p* = 0.001). On the other hand, patients with Cathepsin D-negative tumours, who received tamoxifen therapy, did not have statistically different survival rates as patients who had not received the drug (Fig. 3).

### Clinical outcome of patients with Cathepsin D expressing tumours in relation to HER2

**Fig.4:**
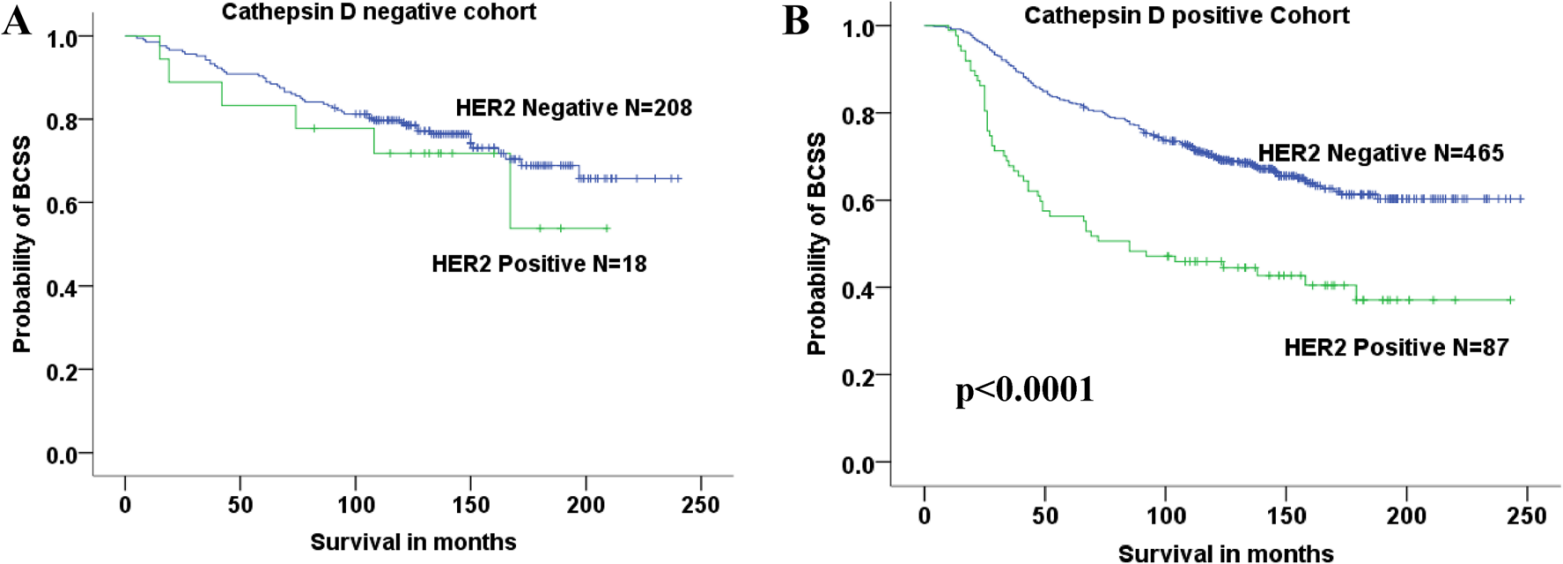
BCSS as a function of Cathepsin D status in subgroups of HER2-negative and HER2-positive patients

BCSS of patients with cancers expressing Cathepsin D was analysed according to HER2 status. Patients with HER2-positive tumours expressing Cathepsin D had significantly lower survival rates than those with negative HER2 (*p* < 0.0001), Fig. 4. In Cathepsin D-negative tumours, HER2 status did not affect BCSS.

## Discussion

In breast cancer, a large number of studies have investigated Cathepsin D expression using either immunohistochemistry or measurement of cytosolic Cathepsin D content (by radiometric immunoassay (IRMA), ELISA or Western blot). Results of immunohistochemical measurement of Cathepsin D expression were variable among different studies, no standard protocols, no control of confounding caused by treatment and inconsistent correlations with prognosis [12]. For this reason, there is a great need to re-evaluate Cathepsin D significance in breast cancer addressing these issues in particular. Göhring et al. concluded that immunohistochemistry (IHC) is the preferred method for measuring Cathepsin D compared to immunoradiometric assays (IRMA) due to its simpler methodology and minimal tissue requirements [19]. In addition, immunohistochemistry provided more predictive data with respect to prognosis. Despite the substantial research on Cathepsin D expression and its prognostic significance in breast cancer, conflicting results have emerged, particularly from studies using immunohistochemistry (IHC) with small patient cohorts. Our study aimed to address this inconsistency by leveraging a large and well-characterized cohort of primary operable invasive breast carcinomas. This well-defined population, with long-term median follow-up and uniform treatment, has previously proven valuable for studying various biomarkers using tissue microarrays.

Our study found that 71.2% of breast cancer tissues displayed positive Cathepsin D expression using the cut-off of > 30% cytoplasmic staining. These results are consistent with findings of other research on Cathepsin D expression in breast cancer [20]. Two studies showed that 68% (17/25) and 58.71% (91/155), respectively, of malignant breast tissues tested had positively stained cells for Cathepsin D [21, 22]. Several approaches, such as immunohistochemistry and cytosolic immunoassay, have demonstrated that in most breast cancers, Cathepsin D is overexpressed 2- to 50-fold compared to its concentration in normal mammary gland cells [23].

A key finding of our study is that Cathepsin D expression significantly correlates with several adverse clinicopathological parameters. Statistical analysis demonstrated a highly significant association of Cathepsin D with poor prognostic variables including high tumour grade (*p* < 0.007) and poor NPI (*p* = 0.002). This was consistent with another study which also found a significant association of Cathepsin D expression with tumour grade (*p* < 0.001) [24]. Unexpectedly, our study did not find a significant correlation between tumour stage and Cathepsin D expression but it showed a trend (*p* = 0.084). No correlation was found between Cathepsin D expression and nodal status in several immunohistochemistry studies in breast cancer [25, 26]. In the available literature, there are conflicting results between different studies regarding this relationship.

Cathepsin D expression was also associated with highly increased risk of subsequent metastasis, recurrence and tumour grade [27, 28]. Prior studies have noted that Cathepsin D is involved in degradation of extracellular matrix and basement membrane in cancers due to its proteolytic activity [29]. A study using a vectorized Cathepsin D inhibitor found that anti-proliferative activity was associated with Cathepsin D inhibition, suggesting that intracellular Cathepsin D plays a major role in cancer cell proliferation [30].

Furthermore, our study found that Cathepsin D expression is a powerful and independent prognostic factor for both breast cancer-specific survival (BCSS) and disease-free interval (DFI). This finding remained significant even after accounting for other key prognostic factors such as lymph node status, tumour size, tumour grade and oestrogen receptor status. These results align with previous research [31–35].

The relationship between Cathepsin D expression and steroid receptor status has been studied by many researchers. This study shows a highly significant association of Cathepsin D expression and ER status (*p* = 0.006) but no association with PgR status. Here, we also show that Cathepsin D is a prognostic marker within ER-positive patients, a group of breast cancer patients with relatively good prognosis. Cathepsin D has subdivided ER+ patients into two prognostic groups. Cathepsin D-negative patients have significantly better BCSS and less risk of either recurrence or metastasis than Cathepsin D-positive patients within this ER+ patient group. Other studies have reported similar findings [11, 31]. While some studies found no association between Cathepsin D expression and ER status, their conclusions may be limited by their small sample sizes and reliance on ELISA for cytosolic Cathepsin D measurement [25, 32].

Our study revealed a highly significant difference in breast cancer-specific survival (BCSS) between patients who received tamoxifen and those who did not, but only in patients with Cathepsin D-positive tumours (*p* = 0.025). This suggests that tamoxifen may increase Cathepsin D concentration in tumours that already express it, thereby altering the prognosis for this specific patient group. Consequently, evaluating Cathepsin D expression in breast cancer prior to initiation of hormonal therapy may predict anti-oestrogen responsiveness. Interestingly, our study and previous research suggest a potential link between Cathepsin D expression and the response to tamoxifen therapy in ER-positive breast cancer patients. Although the previous study did not reach statistical significance (*p* = 0.09), it supports our findings and warrants further investigation [32].

The level of expression of Cathepsin D was correlated with oestrogen receptor status in breast cancer [31]. Studies of RNA levels of Cathepsin D showed that tamoxifen increased Cathepsin D RNA level regardless of the ER status of the tumours and that this increase is directly proportional to protein level in the cytosol in ER+ tumours but not in ER− tumours [33].

Although Cathepsin D is regulated by oestrogen, in ER-negative breast cancer what regulates or stimulates Cathepsin D overexpression is unknown and might involve other pathways, e.g. other enhancers stimulated by transcription factors, hypoxia induced expression and thrombin [1, 34, 35].

The relationship between Cathepsin D and HER2 expression has not been studied extensively. In this study, HER2 expression was highly associated with Cathepsin D expression (*p* = 0.006). Clinical outcome of patients who are Cathepsin D-positive and HER2-positive was compared to HER2-negative patients. Patients with HER2-positive tumours have significantly lower survival rates than those with negative HER2 (*p* < 0.0001). This further emphasizes the association of Cathepsin D with poor prognostic variables. In addition, from this study, Cathepsin D lost its independent significance as prognostic factor (for BCSS) when HER2 expression is introduced in the multivariate analysis along with tumour grade, size and stage.

While previous research has reported an association between Cathepsin D expression and HER2-neu amplification, contradictory findings exist, demonstrating no such link [36, 37]. Additionally, silencing Cathepsin D has been shown to increase ER expression and decrease HER2 expression, suggesting a complex interplay between these proteins [38]. Further investigation into this relationship, including subgroup analysis based on trastuzumab therapy, is warranted to determine the potential of Cathepsin D as a predictor of treatment response.

This study has some limitations. Tumour size, and grade and NPI score information were missing for 25 and 27, respectively, of the 954 cases. While listwise deletion was employed to handle missing data, this could potentially introduce bias into the results. Future studies could explore alternative approaches such as imputation techniques to address missing data and further strengthen the generalizability of findings.

## Conclusion

Cathepsin D expression significantly predicts poor prognosis in breast cancer and is associated with aggressive clinicopathological features and worse outcomes, including shorter breast cancer-specific survival (BCSS), higher risk of recurrence and distant metastasis, suggesting its potential as a prognostic biomarker and therapeutic target.

It is noteworthy that the results suggest a possible interaction between Cathepsin D expression and tamoxifen therapy in ER-positive breast cancer. It could be clinically useful to predict response to tamoxifen treatment and help identify patients at risk of resistance. This observation warrants further investigation to elucidate the underlying mechanisms.

Cathepsin D expression in breast cancer correlates with HER2 status, warranting further investigation in larger, trastuzumab-treated patient cohorts to assess its potential as a predictor of treatment response.

In conclusion, this large-scale study provides compelling evidence for the clinical relevance of Cathepsin D as a prognostic marker in breast cancer. Further research is needed to confirm these findings and to explore the potential therapeutic implications of targeting Cathepsin D in breast cancer treatment.

## Data Availability

Enquiries about data availability should be directed to the authors.
